# Supplementary figures and images for: Role of ELK1 in regulating colorectal cancer progression: miR-31-5p/CDIP1 axis in CRC pathogenesis
Source: PeerJ. 2023 Jul 31;11:e15602. doi: 10.7717/peerj.15602 (PMC10399563; doi:10.7717/peerj.15602)

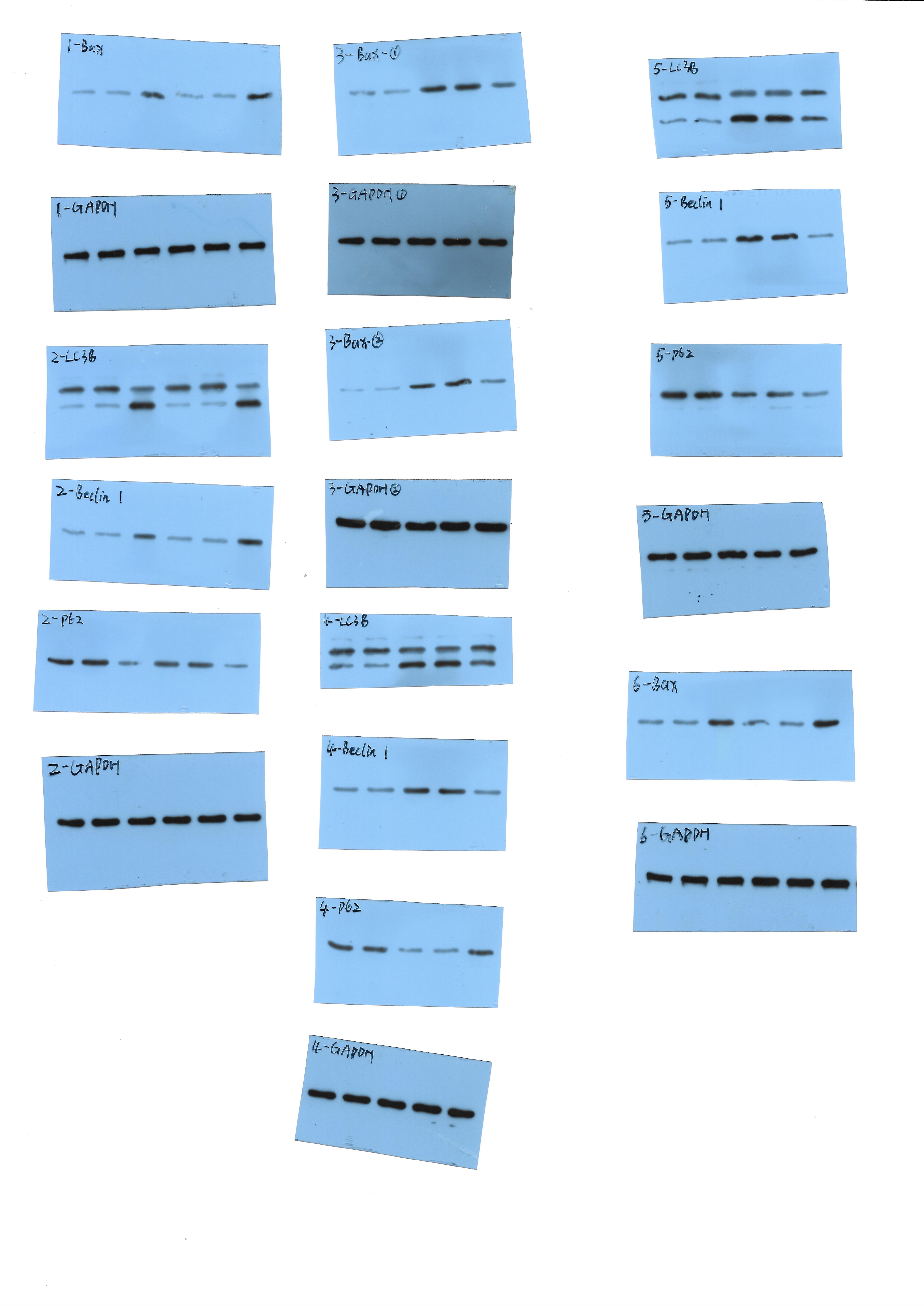

Supplement: Supplemental Information 2 [file peerj-11-15602-s002.zip › original gel/1-6.jpg]

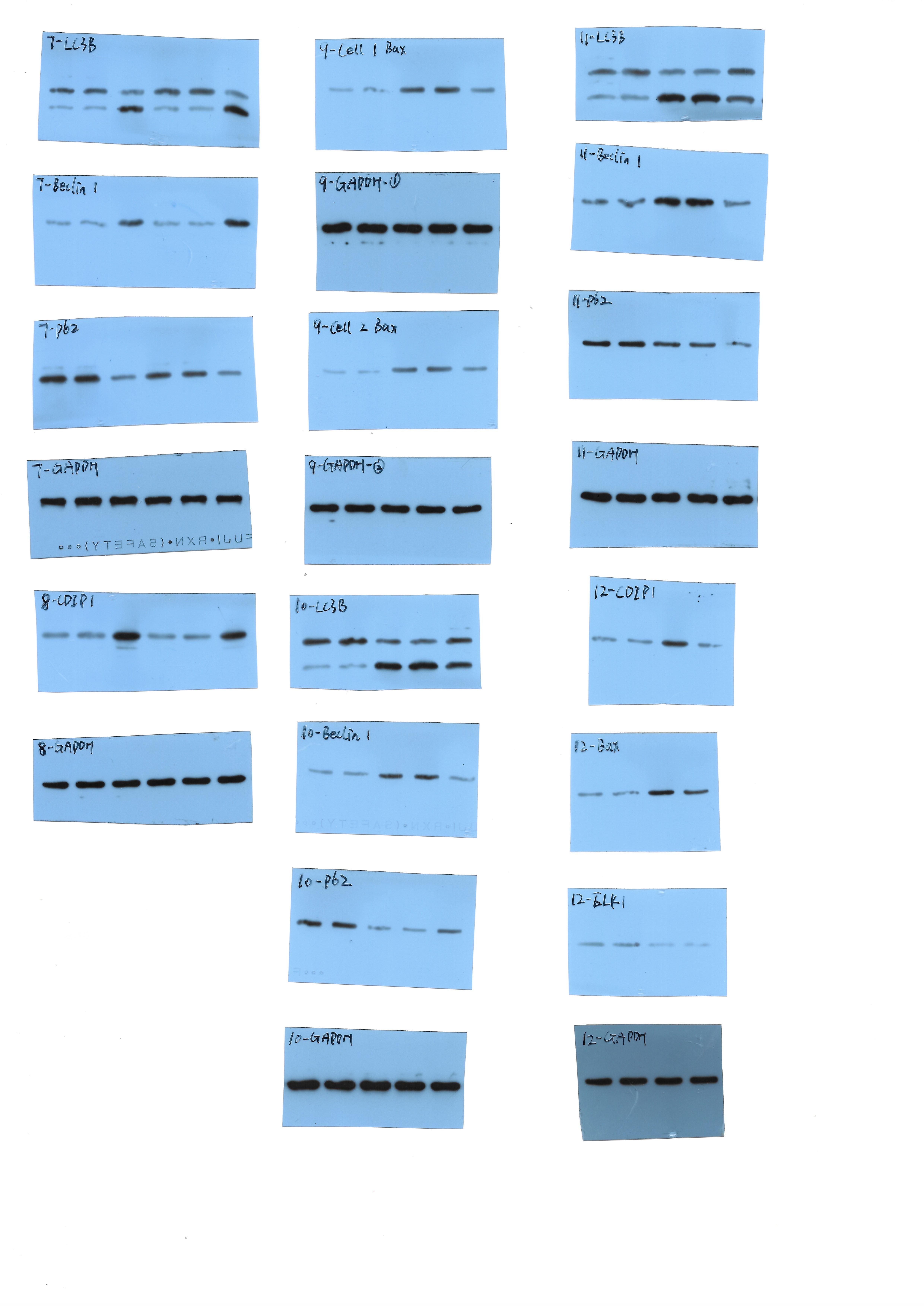

Supplement: Supplemental Information 2 [file peerj-11-15602-s002.zip › original gel/7-12.jpg]

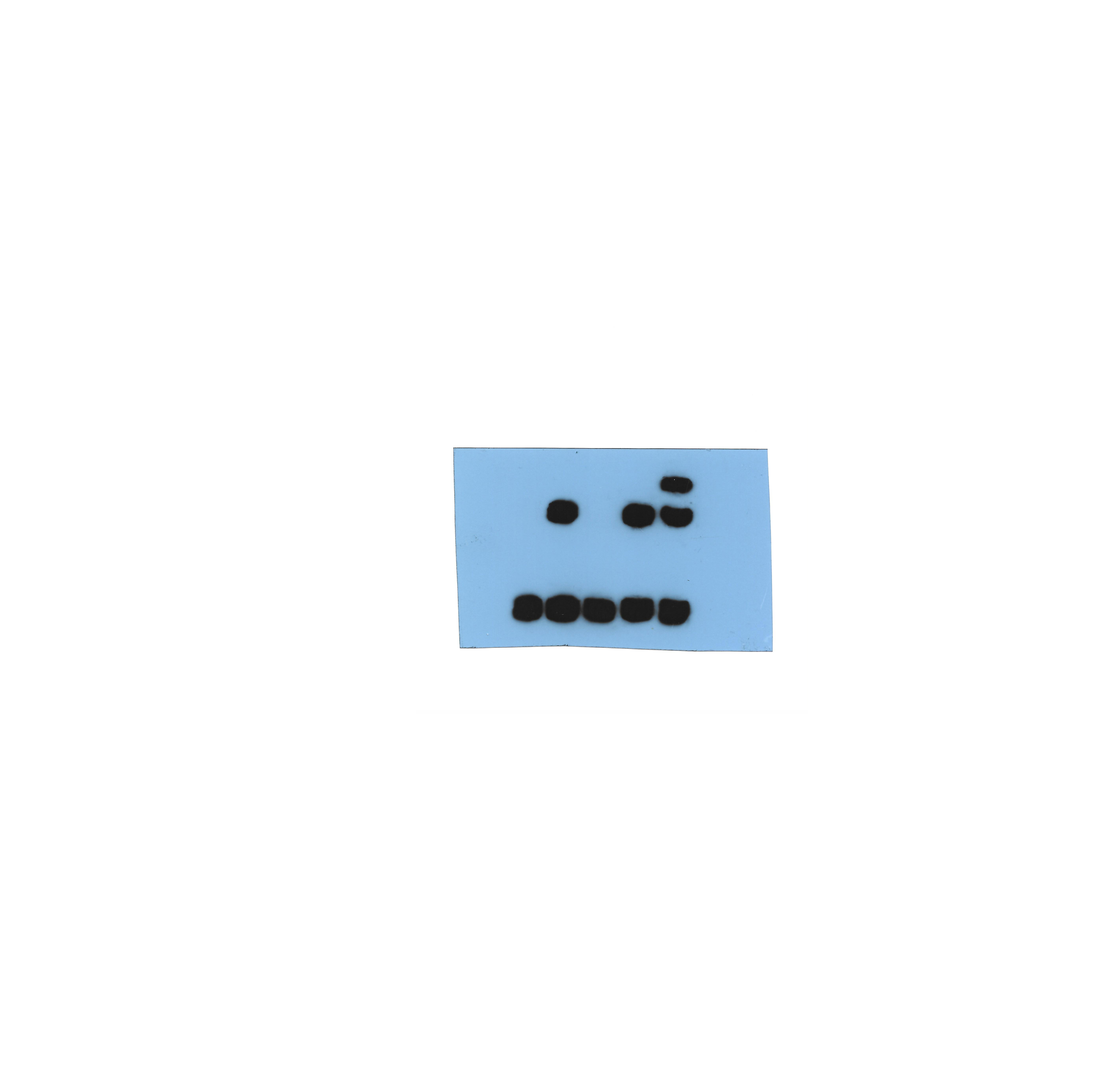

Supplement: Supplemental Information 2 [file peerj-11-15602-s002.zip › original gel/EMSA扫描.jpg]
